# Understanding the Effect of Zein–Resveratrol Interactions on the Mechanical and Functional Properties of Zein Gels

**DOI:** 10.3390/gels12080740

**Published:** 2026-08-18

**Authors:** Iulia Matei, Alexandra Busuioc, Ludmila Aricov, Anca Ruxandra Leonties, Vlad Tudor Popa, Aurica Precupas

**Affiliations:** “Ilie Murgulescu” Institute of Physical Chemistry of the Romanian Academy, 202 Splaiul Independentei, 060021 Bucharest, Romania; iuliamatei@icf.ro (I.M.); alexandra.busuioc01@yahoo.com (A.B.); laricov@icf.ro (L.A.); aleonties@icf.ro (A.R.L.); vtpopa@icf.ro (V.T.P.)

**Keywords:** protein, zein, polyphenol, gel, drug delivery

## Abstract

The potential of zein gels as functional delivery platforms for resveratrol (RESV), a natural polyphenol with antiamyloidogenic action but low systemic bioavailability, is explored by a combination of spectroscopic (circular dichroism, infrared, fluorescence, UV–Vis), calorimetric (differential scanning microcalorimetry) and rheological methods. Molecular docking is also applied for mapping the interaction sites and forces between native zein and RESV. Zein–RESV interactions in solution are systematically evaluated to understand the structural changes from a soluble protein–polyphenol system to a gel network. The concentration-dependent effect of RESV on the structural and thermal stability of zein is investigated in relation to the mechanical and functional properties of zein–RESV gels. It is shown that RESV acts as a promoter of zein aggregation, with a more pronounced effect obtained at a RESV:zein 1:1 molar ratio. By increasing the α-helix content, RESV induces a more ordered zein secondary structure in solution. In gel, the three-dimensional zein network is disrupted by hydrophobic and hydrogen-bonding interactions with RESV. Fluorescence spectra of zein gels point to conformational changes upon interaction that are confirmed by infrared spectroscopy. A significant viscoelastic deformation of the gel occurs when the RESV:zein molar ratio increases to 4:1. The RESV:zein 1:1 gel network demonstrates optimal viscoelastic properties for the release of RESV, following a biphasic transition from diffusion to matrix-controlled release. The findings of this study contribute to the structural design of zein-based systems for encapsulating and delivering bioactive compounds.

## 1. Introduction

The rising need for sustainable, non-toxic biomaterials has intensified research into plant-derived proteins as viable substitutes for animal and synthetic products [1,2]. In this context, zein, the principal storage protein from corn (*Zea mays*), has attracted considerable attention due to its unique physicochemical and functional properties. Zein presents biocompatibility, biodegradability and functional versatility [3], and represents a promising candidate for applications in the food, pharmaceutical and biomedical fields [4].

Structurally, zein is an amphiphilic protein rich in hydrophobic amino acid residues, which accounts for its solubility in aqueous ethanol. It self-assembles into nanospheres and nanofibers [5], a feature that supports its versatility in forming gel-based delivery systems. Recent research reveals that α-zein can form amyloid-like fibrils under low ethanol concentration and near-neutral pH, in weakly alkaline conditions or at 65–75% ethanol [6,7,8]. These characteristics have enabled zein applications in pharmaceutical formulations for the release of active compounds [9], wound dressings [10,11] and edible coatings for the stabilization of sensitive ingredients [12,13]. Despite these advances, the mechanisms governing zein gel formation remain largely unknown. Addressing this gap is essential for the rational design of zein-based systems tailored to specific application requirements.

Polyphenols are well-known for their beneficial effects on human health [14,15]. Their interactions with proteins and other constituents of the food matrices or biological systems can alter their stability, bioavailability and, ultimately, their biological activity [16]. The type and strength of these interactions [17] play a crucial role in determining the structural and functional properties of the resulting systems [18]. Previous studies on the thermal stability of proteins in the presence of polyphenolic compounds reported their potential as aggregation inhibitors using calorimetric and spectroscopic methods, supported by molecular modeling [19,20,21,22]. Interactions between zein and polyphenols occur through both non-covalent and covalent mechanisms [23]. Non-covalent interactions are primarily driven by the affinity between the hydrophobic domains of zein and the aromatic rings of polyphenols. In contrast, covalent interactions occur through oxidative mechanisms, where polyphenols are converted into reactive quinones that bind to nucleophilic amino acids, resulting in stable conjugates with enhanced bioactivity.

This study proposes a mechanistic investigation into how zein–polyphenol interactions in solution influence the self-assembly of zein into polyphenol-containing gels. Resveratrol (RESV, 3,5,4′-trihydroxy-trans-stilbene), a naturally occurring polyphenolic compound belonging to the class of stilbenes, with antiamyloidogenic action but low systemic bioavailability, was selected for this purpose. Our investigation goes beyond routine encapsulation studies to systematically address how zein–RESV molecular interactions in solution directly determine the mechanical properties of the resulting gel network. The novelty of the study lies in using the polyphenol itself as a structural modulator, instead of relying on exogenous enzymes and secondary polymers for structural density [24].

Previous studies have used conventional differential scanning calorimetry (DSC) to evaluate the thermal stability of solid-state zein formulations containing RESV, such as ultrasound-assisted zein nanoparticles or zein–chitosan complex coacervates [25,26]. Similarly, the thermal properties of covalent zein–RESV conjugates have been evaluated in dry forms [27]. These conventional solid-state DSC methods analyze dry thermal transitions (melting or degradation points) and lack the sensitivity required to measure the thermal properties of proteins in dilute liquid environments. This paper employs, for the first time, high-sensitivity microcalorimetry (μDSC) to evaluate the thermal stability of zein in solution and the effect exerted by the presence of RESV. This approach allows for the estimation of thermodynamic parameters of protein aggregation that occurs before the sol-to-gel transition.

Circular dichroism spectroscopy is employed to determine the structural changes produced in zein by increasing concentrations of RESV. Based on these results, two zein gels are prepared, containing different concentrations of polyphenol (RESV:zein molar ratios of 1:1 and 4:1). The RESV:zein molar ratios were selected to compare the effect of an equimolar ratio (1:1) to that of an excess molar ratio (4:1) of RESV on both protein conformation and gel properties. Interactions between zein and RESV within the gel matrices are evaluated by FTIR and fluorescence spectroscopies, with the aim of determining their impact on the mechanical and functional properties of the gels. The rheological behavior of zein and RESV:zein gels is characterized, and the release kinetics of RESV from the 1:1 and 4:1 gels are presented. The gels appear as promising candidates for the sustained, controlled delivery of RESV, due to their hydrophobic character and ability to form compact networks that delay the diffusion of the encapsulated compound. These results are expected to contribute to the development of new zein-based systems to encapsulate, protect and deliver bioactive compounds.

## 2. Results and Discussion

### 2.1. Zein–Resveratrol Interactions in Solution

#### 2.1.1. Conformational Changes in Zein upon Interaction with Resveratrol

**Figure 1:**
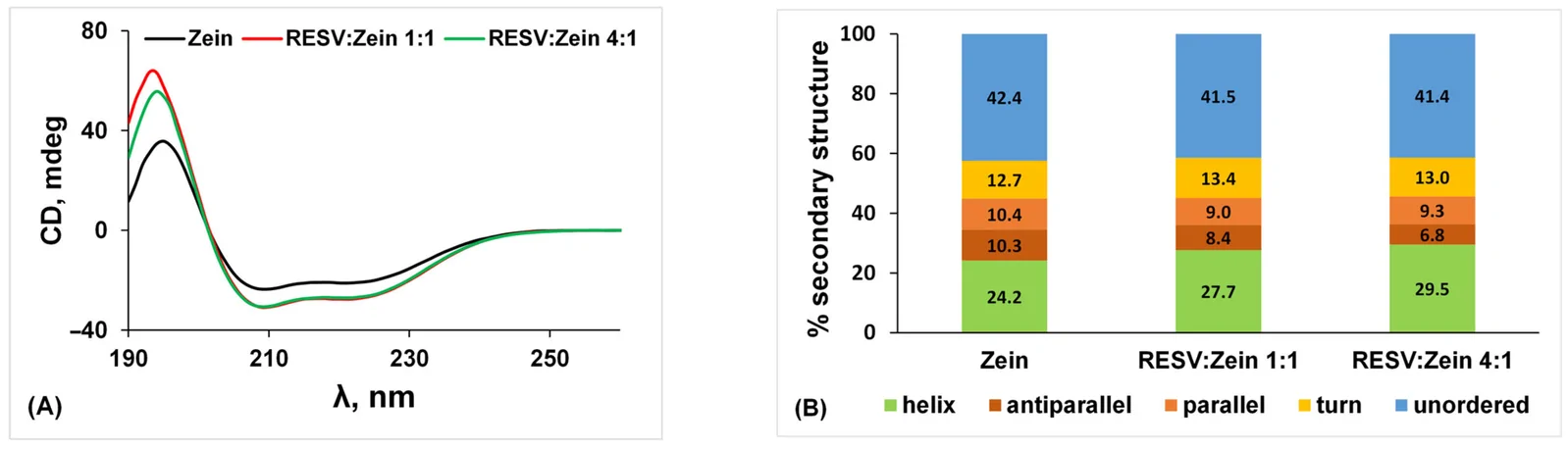
(**A**) Circular dichroism spectra of zein (2 × 10^−5^ M) in the absence and in the presence of RESV at different molar ratios. (**B**) Secondary structure content of zein in the investigated systems.

Circular dichroism spectroscopy allows an estimation of the effect of different concentrations of RESV on the secondary structure of zein. In a typical far-UV circular dichroism spectrum of α-zein (Figure 1A), a strong negative peak at 222 nm (n–π* transition of the peptide backbone) and a strong negative peak at 208 nm coupled with a positive peak at 193–195 nm (π–π* transition of the peptide backbone split by exciton coupling) indicate a predominant α-helix secondary structure [28]. Binding of polyphenols like chlorogenic acid and (–)-epigallocatechin gallate was shown to have a minor effect on the structure of α-zein [29,30].

The secondary structure content of the zein–RESV systems in solution, estimated using the BeStSel web server, reveals an increase in α-helix from 24.2% for native zein to 27.7% and 29.5% at RESV:zein 1:1 and 4:1 molar ratios, respectively (Figure 1B). The increase in α-helix content indicates a more ordered secondary structure of the protein. Since the formation of a protein gel network typically requires the transition from organized structures to unfolded/disordered structures with exposed functional groups [31], these RESV-induced conformational changes are expected to hinder, to some degree, the gelation of zein. The effect of the presence of RESV during the formation of the zein gel network can be accounted for by rheological measurements, as presented in Section 2.3.3. The intermolecular forces responsible for the zein–RESV interaction and subsequent conformational changes can be explored by molecular docking (Section 2.2).

#### 2.1.2. The Effect of Resveratrol on the Thermal Stability of Zein

μDSC measurements were performed to evaluate the effect of the polyphenol on the thermal stability of zein. Previous studies have shown that RESV exerts a dual effect on α-lactalbumin denaturation [32], improves the stability of human serum albumin to thermal unfolding, and modulates the formation of fibrils [33].

**Figure 2:**
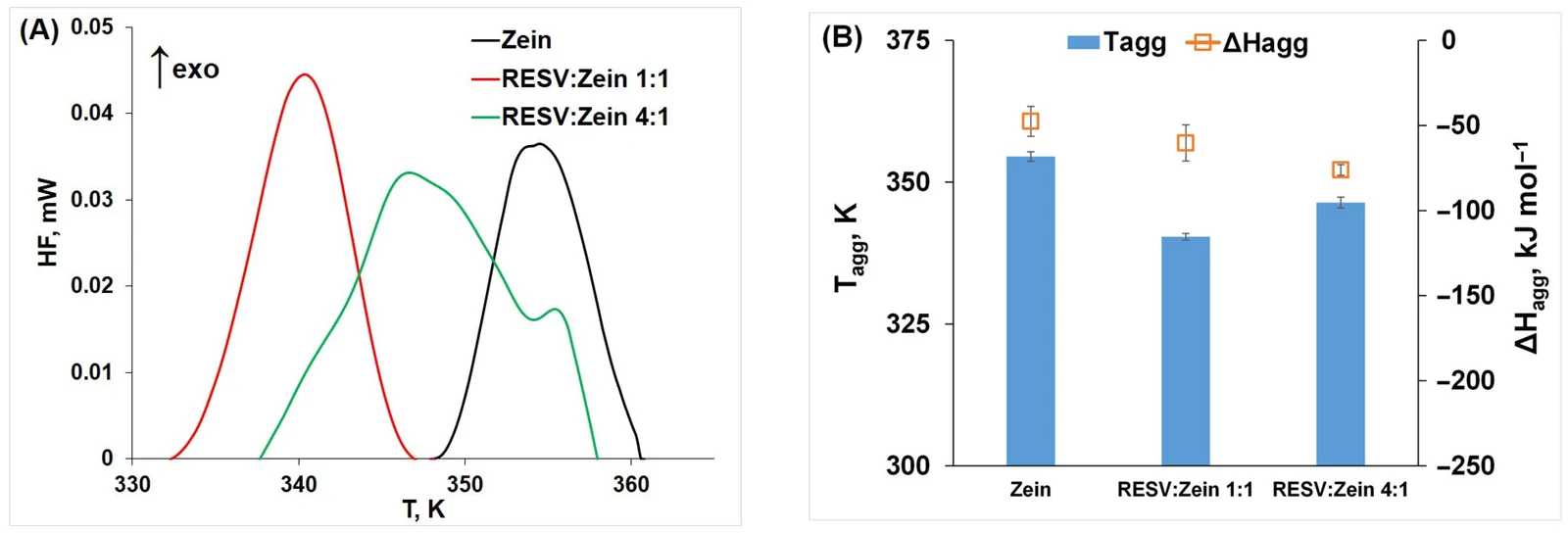
The μDSC thermograms (**A**) and thermodynamic parameters of aggregation (**B**) of zein (6.30 × 10^−4^ M) in the absence and in the presence of RESV at different molar ratios.

The μDSC thermograms of zein and zein–RESV systems are presented in Figure 2A and reveal exothermic peaks corresponding to zein aggregation. As the temperature increases, the gradual unfolding of zein exposes hidden hydrophobic domains, causing conformational rearrangement of the polypeptide chains. Similar exothermic peaks recorded using µDSC have been associated with protein aggregation and self-association in studies on whey proteins [34], serum albumin [35] and hemoglobin [19,21]. The similar peak shape observed for the zein and RESV:zein 1:1 systems changes to a broader peak with a shoulder at higher temperature for RESV:zein 4:1, suggesting that the excess of polyphenol induces different aggregate formation.

The increasing temperature induces protein unfolding, with solvent exposure of the hydrophobic domains and subsequent aggregation. The thermodynamic parameters of this process (aggregation temperature, T_agg_, and enthalpy change, ΔH_agg_) are shown in Figure 2B and indicate a concentration-dependent effect of RESV on protein aggregation. The overall lower T_agg_ values obtained in the presence of RESV point out its role as a promoter of zein aggregation, with a higher effect obtained at a RESV:zein 1:1 molar ratio. The higher values of |ΔH_agg_| obtained with increasing RESV concentration signify a stronger destabilizing effect of the polyphenol on the unfolded protein structure.

### 2.2. Molecular Docking of Resveratrol to Zein

**Figure 3:**
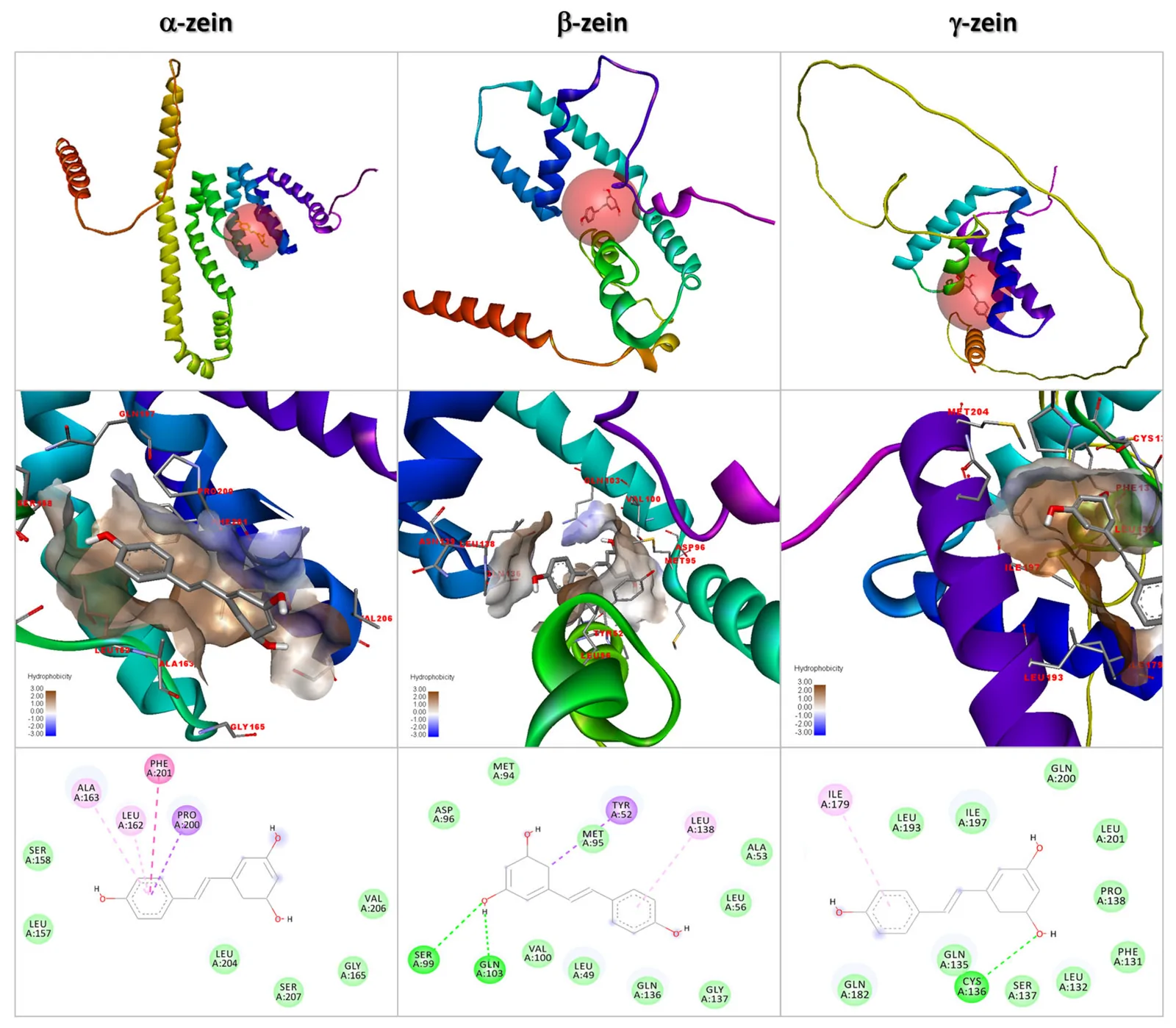
Molecular docking of RESV to α-, β- and γ-zein isoforms. (**Top row**) Docking poses with the lowest interaction energy (RESV and zein are rendered in stick and solid ribbon representations, respectively). (**Middle row**) Hydrophobic cavities of the RESV interaction sites with zein isoforms. (**Bottom row**) Two-dimensional diagrams depicting the amino acid residues located at a distance less than 5 Å from RESV; the dashed lines represent intermolecular interactions of different origins: π-sigma (purple), π-alkyl (light pink), π-π (pink), hydrogen bond (dark green), van der Waals (light green).

The interaction of RESV with three isoforms of zein (α, β, and γ) was investigated by molecular docking. Interaction energies of −6.5 kcal/mol (α-zein), −6.2 kcal/mol (β-zein), and −6.4 kcal/mol (γ-zein) were predicted for the most stable docking poses depicted in Figure 3. The main intermolecular interactions of RESV with α-zein are hydrophobic in nature and occur with amino acid residues Pro200 (3.97 Å, π-sigma interaction), Leu162 (4.74 Å, π-alkyl), Phe201 (4.93 Å, π-π T-shaped) and Ala163 (5.03 Å, π-alkyl). For β-zein, the docking results predict hydrophobic interactions between RESV and the Tyr52 (3.76 Å, π-sigma) and Leu138 (5.31 Å, π-alkyl) residues, but also hydrogen bonding with Ser99 (3.13 Å) and Gln103 (2.13 Å). For γ-zein, a hydrophobic interaction with Ile179 (5.39 Å, π-alkyl) and a hydrogen bond with Cys136 (3.14 Å) are identified.

These computational results reveal the preference of RESV for zein hydrophobic pockets rich in Leu, Phe, Ala, Pro and Tyr amino acids. This accounts for the significant effect exerted by RESV upon zein’s secondary structure. The polyphenol phloretin, belonging to the dihydrochalcone class, was similarly found to have affinity for a hydrophobic pocket of zein lined with Leu, Ala and Tyr residues [36]. We also note hydrogen bonding interactions between RESV and amino acids Gln, Ser and Cys. The molecular docking analysis therefore reveals that hydrophobic interactions and hydrogen bonds are the primary driving forces of the zein–RESV association.

### 2.3. Characterization of Zein-Based Gels for Resveratrol Encapsulation and Release

#### 2.3.1. Conformational Changes in Zein upon Gelation and Interaction with Resveratrol

**Figure 4:**
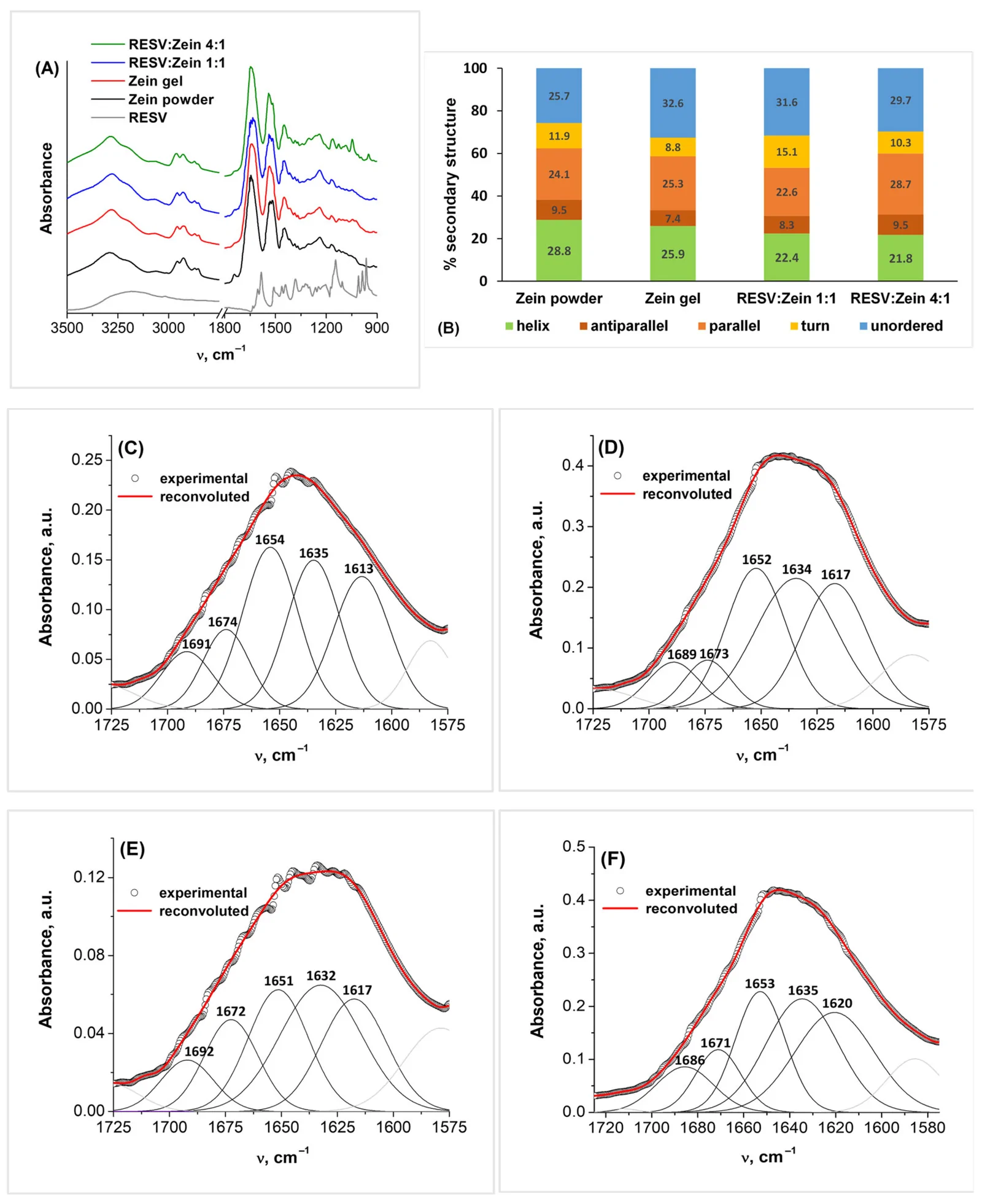
(**A**) FTIR spectra of zein, RESV:zein 1:1 and RESV:zein 4:1 gels, and of zein and RESV powders. (**B**) Secondary structure content of zein in the investigated systems. (**C**–**F**) Deconvolution of the amide I spectral region for zein powder (**C**), zein gel (**D**), RESV:zein 1:1 gel (**E**) and RESV:zein 4:1 gel (**F**).

FTIR spectroscopy was employed to examine the interactions that occur between zein and RESV within the gel matrix and to assess the extent of structural alterations induced. Figure 4 displays the FTIR spectra of zein gel, RESV:zein gels prepared at 1:1 and 4:1 molar ratios, and of zein and RESV powders as references. The characteristic signals of the main functional groups were identified by referring to literature data on zein [37,38] and resveratrol [39,40].

For RESV, the broad band with a maximum at 3175 cm^−1^ is ascribed to O-H stretching vibrations. The weak band observed at 3019 cm^−1^ can be attributed to =C–H stretching of aromatic and vinyl bonds. In the 1610–1500 cm^−1^ range, the spectrum exhibits characteristic bands of the conjugated π-electron system, ascribed to aromatic C=C stretching (1605, 1509, 1463, 1441 cm^−1^) and olefinic C=C stretching (1584 cm^−1^) vibrations. Two strong bands observed at 1382 and 1145 cm^−1^ can be assigned to in-plane bending of phenolic O-H groups and to stretching of C-O groups, respectively.

The two intense bands at 1010 and 964 cm^−1^ are characteristic of the trans-RESV configuration and correspond to out-of-plane bending vibrations of the =C–H bonds attached to the trans-olefinic bond. A strong band observed at 829 cm^−1^ can be attributed to deformation vibrations of the C-H bonds in the benzene rings. In the RESV:zein 4:1 gel system, these three bands shift to 1013, 951 cm^−1^ and 838 cm^−1^. This indicates a change in the microenvironment of the extended π-system of RESV, due to hydrophobic interactions with the zein matrix. The in-plane bending vibration of the phenolic O-H groups of RESV is also shifted to higher wavenumbers upon interaction, from 1339 cm^−1^ to 1342 cm^−1^, confirming that the polyphenol’s hydroxyl groups actively engage in hydrogen bonding with the protein. In the RESV:zein 1:1 gel, most of the characteristic bands of RESV are absent, demonstrating efficient encapsulation within the hydrophobic zein matrix, which limits the bending and stretching of various RESV bonds [38].

Regarding the FTIR spectrum of zein, in the following we analyze the positions and intensities of its characteristic vibrational modes in order to assess the extent of conformational changes induced upon gelation and interaction with RESV.

The amide A band of zein arises from N-H and O-H stretching vibrations of the polypeptide chain. Its position is slightly changed upon gelation, with a shift in the band maximum from 3290 cm^−1^ in zein powder to 3282 cm^−1^ in zein gels. This is indicative of the formation of a more extended intermolecular hydrogen bonding network upon gelation.

In the 3000–2800 cm^−1^ spectral range, associated with C–H stretching vibrations of the CH_2_ and CH_3_ groups, variations in the relative intensity of the bands at 2927 and 2958 cm^−1^ are noted following interaction with RESV. Also, these bands shift to 2930 and 2962 cm^−1^ in the RESV:zein 4:1 gel sample. The intensity ratio of the two bands changes from A_2927_/A_2958_ = 1.03 in zein gel to A_2930_/A_2962_ = 0.97 in RESV:zein 4:1 gel. These variations indicate changes in the microenvironment of the hydrophobic regions of zein, further confirming the role played by hydrophobic forces in the zein–RESV interaction.

The amide I region (1700–1600 cm^−1^) comprises C=O stretching (~80%), N-H bending, and C-N stretching (~20%) vibrations of the polypeptide chain, correlated to the protein backbone conformation [41]. Within this spectral region, the secondary structure components of zein absorb as follows: β-sheet antiparallel (1690–1682 cm^−1^), β-turn (1682–1662 cm^−1^), α-helix (1665–1645 cm^−1^), random coil (1645–1635 cm^−1^) and β-sheet parallel (1635–1610 cm^−1^). By deconvoluting the amide I band into these five components (Figure 4C–F and Table S1), we can estimate the secondary structure content of zein in the investigated systems. We note no significant shifts in the position of the five bands, but the changes in their relative contributions account for a decrease in the α-helix content of zein upon gelation (from 28.8% in zein powder to 25.9% in zein gel), and a further decrease in the gel matrix containing RESV (21.8% for RESV:zein 4:1) (Figure 4B).

The amide II spectral region (1600–1500 cm^−1^) reveals mostly C-N and C-C stretching vibrations in the 1540–1520 cm^−1^ range. The amide III band (1330–1220 cm^−1^) reveals C-N and C-C stretching, and N-H and C-H bending vibrations. These distinctive protein bands are consistently present across all zein samples and maintain comparable positions subsequent to interaction with RESV. The lack of notable band shifts is consistent with the relatively small secondary structure changes determined. Further spectral alterations are observed in the 1450–900 cm^−1^ range, where the distinctive vibrations of aromatic and phenolic groups intensify with the high RESV concentration in the RESV:zein 4:1 gel.

#### 2.3.2. Emissive Properties of Zein Gels Containing Resveratrol

**Figure 5:**
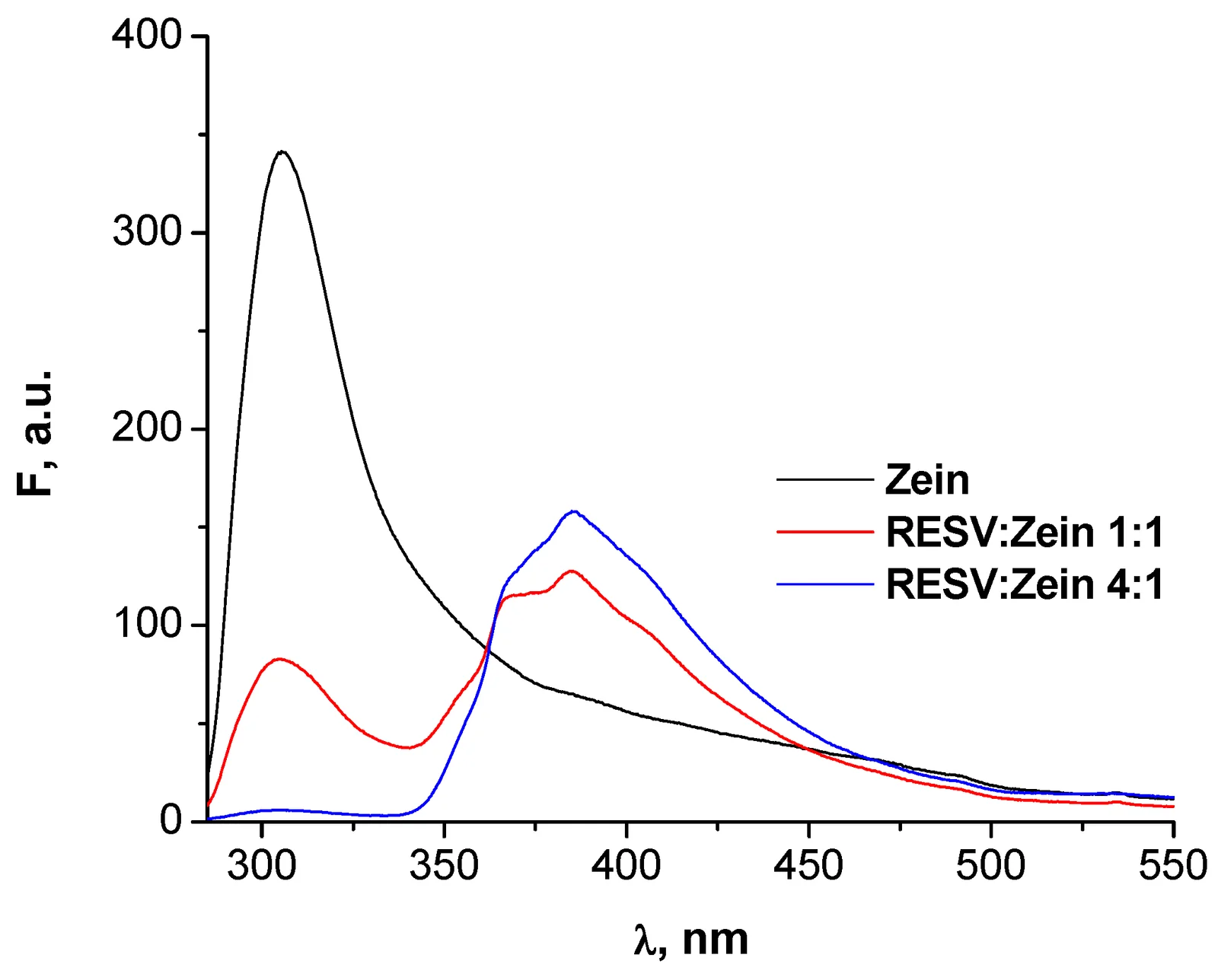
Fluorescence spectra of zein, RESV:zein 1:1, and RESV:zein 4:1 gels.

Fluorescence spectroscopy provides insight into the microenvironment surrounding the fluorescent amino acid residues of zein (Tyr, Phe) within the gel networks. The position of the emission band of zein in the gel state (306 nm, Figure 5) undergoes a slight hypsochromic shift in the presence of RESV (304 nm). This effect is consistent with exposure to hydrophobic RESV molecules and with conformational changes in the protein upon interaction, as revealed by infrared spectroscopy. The emission of zein is strongly quenched by RESV in a concentration-dependent manner. At RESV:zein molar ratio of 4:1, the excess of polyphenol determines high local concentrations of quencher in the vicinity of the emissive amino acid residues, resulting in the strong quenching observed. The absorption spectrum of RESV displays a maximum at 321 nm [42], which significantly overlaps the emission spectrum of zein (Figure S1 in the Supplementary Material). Consequently, the increased proximity of the RESV molecules enhances the possibility of quenching by Förster resonance energy transfer [43], leading to the almost complete disappearance of the fluorescence band of zein, as observed in Figure 5.

In RESV:zein 1:1 and RESV:zein 4:1 gels, we also note the appearance of a fluorescence band with maxima at 367 and 385 nm, and a shoulder at 405 nm. This emissive pattern is consistent with the coexistence in the gel samples of trans-RESV (emission maximum at 400 nm [44]) and cis-RESV (emission maxima at 367 and 385 nm [45]). Cis-RESV is produced by photoisomerization under the UV irradiation at 280 nm used to excite the samples [45].

#### 2.3.3. Rheological Behavior of Zein Gels Containing Resveratrol

**Figure 6:**
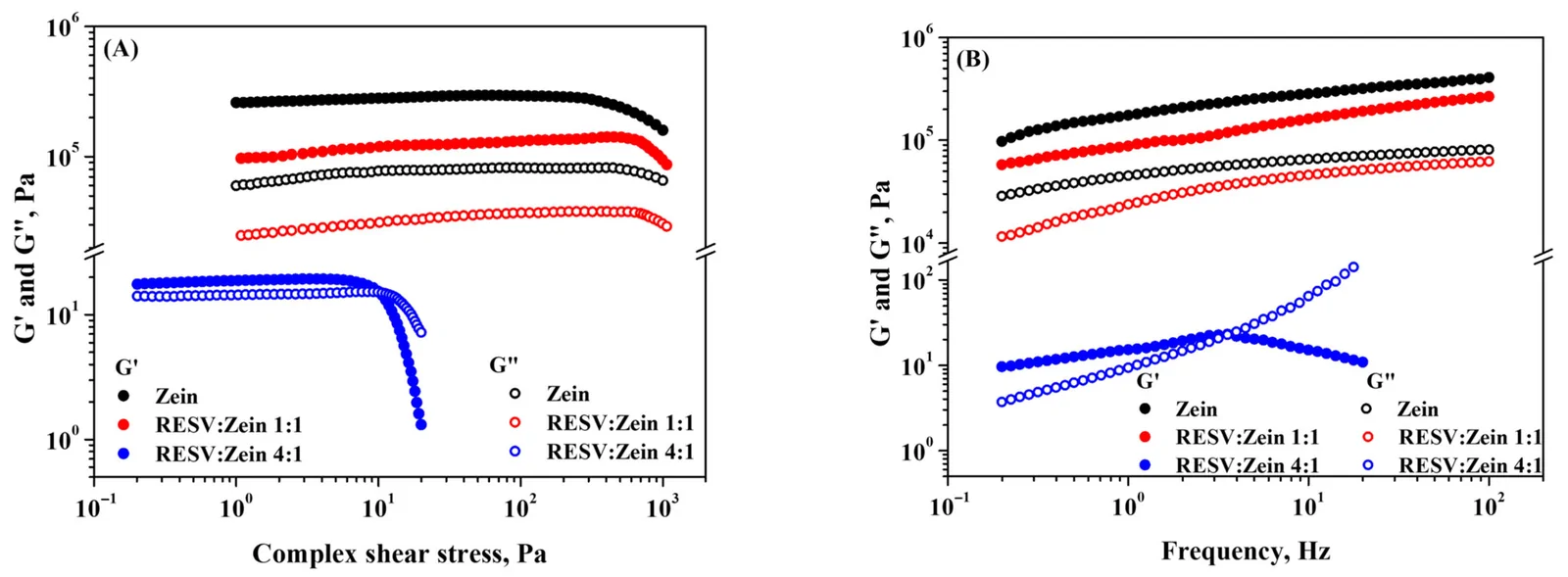
Variation in the viscoelastic moduli G′ and G″ with (**A**) complex shear stress and (**B**) frequency for zein, RESV:zein 1:1 and RESV:zein 4:1 systems, at 298 K.

The viscoelastic characteristics of zein and RESV:zein systems at the two molar ratios, 1:1 and 4:1, were examined by amplitude (Figure 6A) and frequency (Figure 6B) analyses.

Figure 6A presents the relationship between the viscoelastic moduli (G′—storage modulus and G″—loss modulus) and the complex shear stress at a constant frequency of 1 Hz. Up to a shear stress of approximately 200 Pa, the zein and RESV:zein 1:1 systems demonstrate elastic rheological behavior (G′ > G″) within the linear viscoelastic region (LVER), suggesting a wide range of LVER. The presence of RESV at a 1:1 ratio leads to a decrease in both viscoelastic moduli, whereas the deformation characteristics of the sample remain unaffected. Zein forms a three-dimensional interconnected network through strong hydrophobic interactions and hydrogen bonds among its amino acid chains [46]. Upon addition of small hydrophobic molecules of RESV, the self-assembly of zein is disrupted by new interactions between the polyphenol and the hydrophobic domains of the protein, which accounts for the observed reduction in rheological moduli.

A significant viscoelastic deformation occurs when the RESV:zein ratio is increased to 4:1. The LVER and viscoelastic moduli values show a significant decrease, up to four orders of magnitude lower than the zein system. In addition, a yield point is observed at a low shear stress of approximately 10 Pa, where G′ becomes lower than G″, which corresponds to a plastic deformation and a transition to a viscoelastic fluid-like state. The rheological behavior of RESV:zein at a molar ratio of 4:1 is attributed to the excess of polyphenol. At an equimolar ratio of 1:1, the gel network exhibits increased flexibility without deforming. In contrast, an excess of polyphenol at a ratio of 4:1 results in the loss of the strong, stable and structured gel characteristics of the system, leading to a transition to a soft gel that exhibits liquid-like flow properties. This is also visible in the tube inversion test (Section 4.2.4).

Figure 6B depicts the relationship between the viscoelastic moduli and the frequency at constant shear stress from LVER. The rheograms obtained validate the amplitude stress measurements. For the zein and 1:1 RESV:zein systems, G′ consistently exceeded G″ over the entire applied frequency range, which exhibited a weak frequency dependence, characteristic of a stable gel. In the 4:1 RESV:zein system, a frequency dependence is observed, with a crossover point (G′ = G″) at approximately 3.5 Hz, which corresponds to the relaxation time of the sample. At frequencies below 3.5 Hz, the observed behavior resembles that of a viscoelastic solid, characterized by G′ > G″. An opposite behavior was observed above the recorded critical frequency, where G″ exceeded G′, indicating a viscoelastic liquid character.

### 2.4. Resveratrol Release from Zein Gel

The release of the polyphenol from the RESV:zein 1:1 and 4:1 gels, in 20 mM phosphate buffer at pH 7.4, was evaluated at physiological temperature (310 K) using different kinetic models [47]. The initial release phase (0–5 h) was best described by the first-order kinetic model, whereas the Weibull model provided the best overall fit. The Weibull model was also employed to simulate the in vitro release mechanism of curcumin in a nanohydrogel composed of whey protein isolate nanofibers and pectin [48]. The fitting analysis of RESV release from zein gels using the Higuchi and Korsmeyer–Peppas models for the initial release phase is included in the Supplementary Material (Figures S2 and S3).

For the RESV:zein 1:1 gel, the release profile presents a maximum cumulative release of 18.82% after 38.4 h, characterized by a relatively fast initial release during the first 5 h, followed by a plateau phase where the release rate significantly decreases. The plateau phase can be attributed to the intrinsic insolubility of zein in the aqueous release medium. Due to the poor aqueous solubility and hydrophobic nature of zein, exposure to the phosphate buffer promotes structural rearrangement and matrix compaction. As the gel structure becomes denser, diffusion is restricted, leading to the entrapment of a substantial fraction of RESV within the hydrophobic core of the gel. Thus, the overall release involves a two-phase process: the initial concentration-driven diffusion followed by matrix-controlled release.

**Figure 7:**
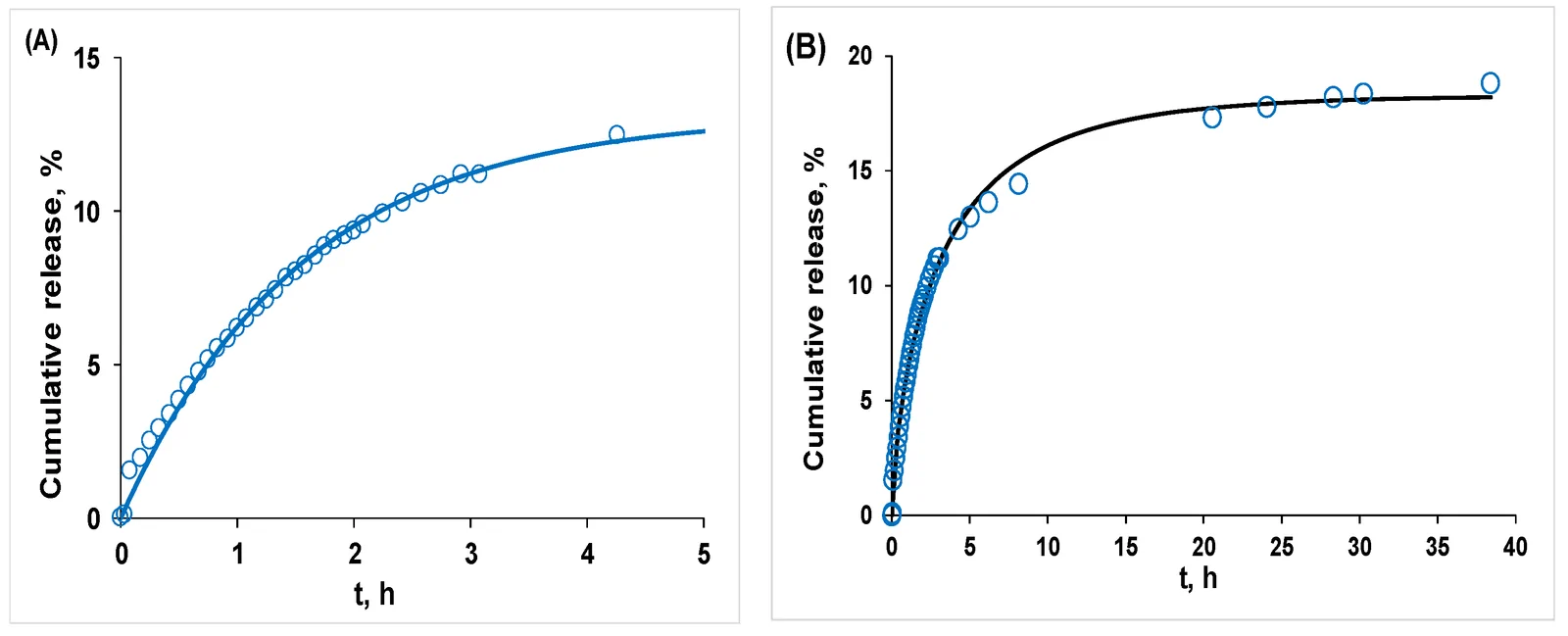
Cumulative percentage release of RESV from the RESV:zein 1:1 gel, in 20 mM phosphate buffer at pH = 7.4 and 310 K: (**A**) initial release phase (0–5 h) described by a first-order kinetic model; (**B**) best fit using the Weibull model.

The initial release phase (0–5 h) is well-described by a first-order kinetic model (Equation (1), Figure 7A) yielding a release rate constant *k* = 0.645 ± 0.020 h^−1^ (coefficient of determination R^2^ = 0.994) that corresponds to a release half-life *t*_1/2_ = 1.08 h. The subsequent plateau phase deviates from the first-order behavior. As such, the overall release mechanism cannot be described by a simple first-order model and is more accurately represented by the Weibull Equation (2), which accounts for both the initial diffusion-controlled release and the plateau phase. The Weibull model provides the best overall fit (R^2^ = 0.995, Figure 7B) to the experimental data, yielding a maximum theoretical release of 18.28%, τ = 2.35 ± 0.04 h^β^ and β = 0.70 ± 0.02. The value of β < 1 indicates a release process characterized by a progressively decreasing release rate, which is characteristic of diffusion-controlled systems exhibiting increasing resistance to mass transfer over time. This is consistent with the structural evolution of the zein gel in the aqueous medium, where water acts as a non-solvent and induces matrix compaction. Consequently, RESV diffusion becomes progressively restricted, leading to the observed asymptotic release behavior. The release value predicted by the model (18.28%) is similar to the experimental plateau (18.82%), indicating that a large fraction of RESV remains entrapped within the hydrophobic zein.

This limited release represents an advantage for oral delivery. Without digestive enzymes, the gel matrix acts as a barrier that prevents early RESV leakage and burst release. This keeps the remaining ~81% of the polyphenol protected from degradation during transit. After ingestion, this trapped fraction will be slowly released as gastrointestinal enzymes break down the zein matrix, changing the process from slow diffusion to complete, enzyme-triggered release.

**Figure 8:**
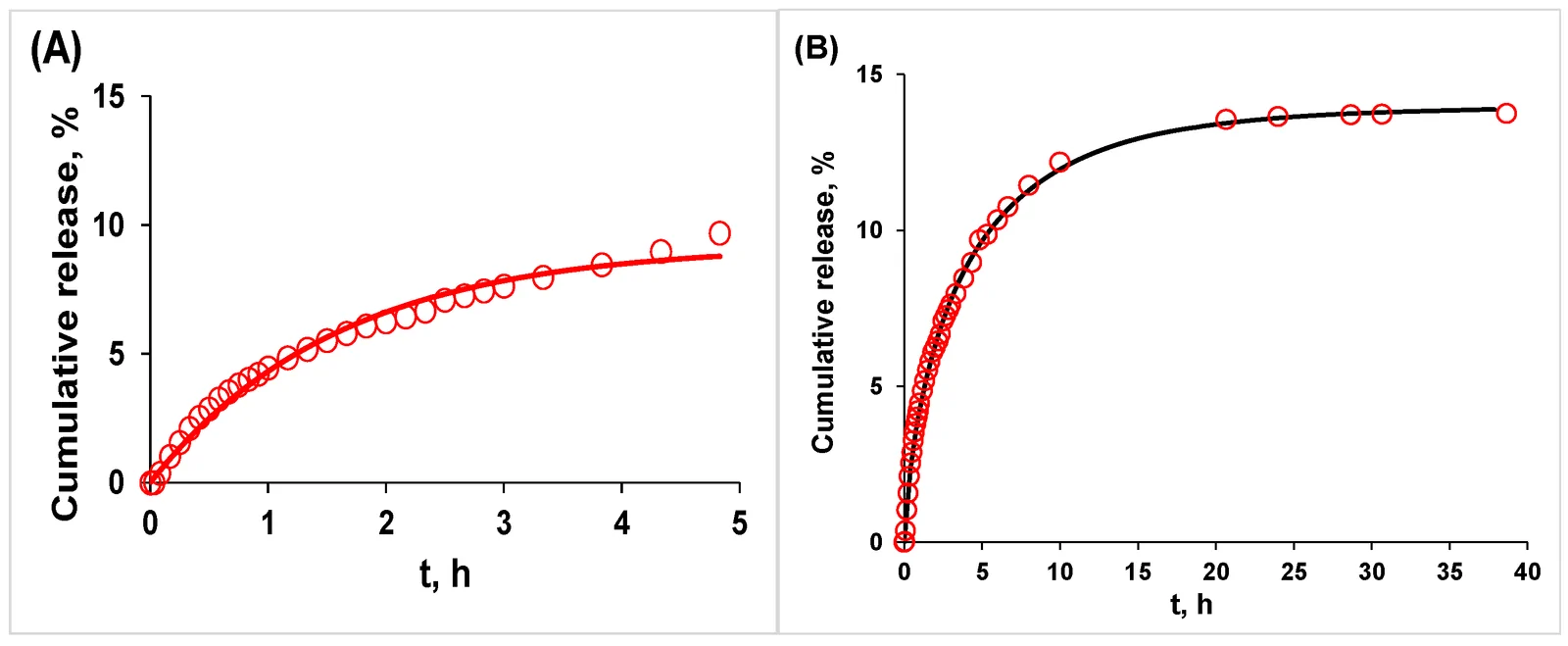
Cumulative percentage release of RESV from the RESV:zein 4:1 gel, in 20 mM phosphate buffer at pH = 7.4 and 310 K: (**A**) initial release phase (0–5 h) described by a first-order kinetic model; (**B**) best fit using the Weibull model.

For the RESV:zein 4:1 gel, the initial phase (0–5 h) follows first-order kinetics with a release rate constant k = 0.630 ± 0.035 h^−1^ (R^2^ = 0.986) that corresponds to a release half-life *t*_1/2_ = 1.10 h (Figure 8A). At 1:1 and 4:1 molar ratios, both gel systems exhibit nearly identical initial release rate constants, showing that RESV diffusion drives the first 5 h of release irrespective of the initial RESV loading. Nevertheless, the 4:1 gel reaches a lower cumulative release (9.23%) during this early phase than the 1:1 gel (13.00%).

After 5 h, water-induced zein matrix compaction causes the release from the 4:1 gel to deviate from first-order kinetics, making the Weibull model a better fit for describing the entire 40 h release profile (Figure 8B). The model provides a good fit with τ = 2.72 ± 0.03 h^β^ and β = 0.72 ± 0.01 (R^2^ = 0.998), confirming that RESV release is governed by a coupled process involving simultaneous diffusion through the gel network and compaction of the protein chains. The maximum theoretical release predicted by the model is 13.96%, revealing a larger fraction of RESV entrapped within the 4:1 gel (86%) compared to the 1:1 gel (81%). The similar values for the Weibull shape parameter β in both systems (0.70 for the 1:1 gel and 0.72 for the 4:1 gel) confirm that while the higher RESV concentration in the 4:1 matrix restricts the long-term delivery capacity (13.96% vs. 18.28%), it does not alter the transport mechanism governed by progressive matrix compaction.

By comparison to our formulations, the release of RESV from core–shell zein–bovine serum albumin nanoparticles occurs with a cumulative release of 98% after 3.5 h, indicating weak interactions between RESV and the modified zein nanoparticles [49]. The release profile of RESV from nanoparticles of zein and alendronate-crosslinked zein reveals a more complex, diffusion-controlled pattern, with an initial burst stage (26–43% cumulative release, depending on the nanoparticle formulation) followed by a zero-order stage and a plateau after 7 h (44–49% cumulative release) [50].

The release profile obtained in physiological buffer highlights a controlled, diffusion-driven mechanism governed by matrix compaction. In the context of oral delivery, this slow diffusion behavior suggests a highly protective potential within the gastrointestinal tract. Due to its dense hydrophobic structural network, zein exhibits native resistance to pepsin degradation and structural degradation in simulated gastric fluid [51]. In such acidic environments, the medium typically promotes further matrix compaction, which would minimize premature RESV leakage. Conversely, upon reaching the simulated intestinal fluid, the combination of a neutral pH and the catalytic action of pancreatic enzymes (such as pancreatin) is known to trigger gradual matrix erosion [52]. These findings indicate that the RESV:zein gels have the potential to function as a dual-controlled system: protecting the bioactive compound from gastric degradation and ensuring a sustained, enzyme-driven release directly within the intestinal tract to optimize oral bioavailability.

While long-term shelf-life studies remain a subject for future optimization, the spectroscopic and rheological profiles indicate high structural integrity during storage. The hydrophobic interactions and hydrogen bonding within the RESV:zein gels entrap the hydroalcoholic solvent, mitigating phase separation or syneresis over time. Furthermore, the accommodation of RESV within the internal hydrophobic pockets of zein, as validated by fluorescence and molecular docking, serves as a protective shield against light and ambient oxygen, a feature commonly exploited in zein-based protective delivery systems.

To turn these structural findings into real healthcare or food products, future research will focus on testing biological performance. This will include the retained antioxidant capacity of RESV, evaluating cell safety and uptake, and running in vivo pharmacokinetic trials to confirm improved concentration of RESV in the bloodstream.

## 3. Conclusions

This study provides new insight into the interactions of zein with RESV and their involvement in the development of protein gel structures. The transition from protein–polyphenol systems in solution to three-dimensional gel networks is governed by non-covalent interactions and protein conformational changes, as circular dichroism and FTIR results indicated. Molecular docking confirmed that hydrophobic interactions and hydrogen bonds are the predominant driving forces governing RESV association with native zein.

The formation of the zein gel network is driven by strong hydrophobic interactions and hydrogen bonding among amino acid residues. RESV modulates zein self-assembly by interacting with the protein’s hydrophobic domains, disrupting intermolecular associations and resulting in lower rheological moduli at an elevated RESV:zein 4:1 molar ratio. These findings contribute to a better understanding of the molecular mechanisms underlying the sol–gel transition and offer valuable information for the rational design of zein-based delivery systems with tailored structural and functional properties.

The release of RESV from the RESV:zein 1:1 gel involves two phases. The initial rapid availability of the bioactive compound is followed by prolonged retention driven by the compaction of the hydrophobic zein matrix in aqueous buffer. While the in vitro release stabilizes at ~19% for the RESV:zein 1:1 gel and at ~14% for the RESV:zein 4:1 gel, the remaining fraction of polyphenol is hypothesized to be progressively released in vivo, through enzymatic degradation of the zein matrix [52] within the intestinal tract. Consequently, the RESV:zein gels represent highly promising candidates for subsequent investigations conducted in simulated digestive fluids. Given that zein maintains a native structural resistance in gastric environments but is highly susceptible to pancreatin digestion, evaluating the erosion of the protein matrix in future trials could confirm whether this dual-controlled mechanism (diffusion-limited gastric retention coupled with enzyme-triggered intestinal erosion) enables complete polyphenol release. This approach offers a valuable perspective for designing functional delivery platforms aimed at enhancing the oral bioavailability of RESV within the gastrointestinal tract.

## 4. Materials and Methods

### 4.1. Materials

Zein (Z3625, protein content > 80%) and trans-resveratrol (purity > 98%) were purchased from Sigma-Aldrich (Saint Louis, MO, USA) and Evolva SA (Reinach, Switzerland), respectively. Ethanol (≥99.5%) and sodium hydroxide (>99%) were purchased from Chimreactiv SRL (Bucharest, Romania) and Lach-ner (Neratovice, Czech Republic), respectively. An 80% (*v*/*v*) ethanol aqueous solution or a pH 11 sodium hydroxide 5 mM solution with 1% ethanol (*v*/*v*) was used to solubilize zein and prepare the working samples, in accordance with literature protocols [53,54].

### 4.2. Sample Preparation and Instrumentation

#### 4.2.1. Circular Dichroism Spectroscopy

Solutions of zein and RESV:zein at molar ratios of 1:1 and 4:1 were prepared in 80% (*v*/*v*) aqueous ethanol. The zein concentration was maintained constant at 2 × 10^−5^ M. The circular dichroism spectra of RESV at 2 × 10^−5^ M (corresponding to a 1:1 molar ratio) and at 8 × 10^−5^ M (corresponding to a 4:1 molar ratio) were subtracted from the corresponding RESV:zein spectra. Samples were analyzed at 298 K, in a wavelength range of 190–260 nm, using a JASCO J-815 CD spectropolarimeter (JASCO Corporation, Tokyo, Japan). A 1 nm bandwidth, 0.05 cm path length, 100 nm/min scanning speed, and 3 accumulations were used to record each spectrum.

The degree of zein unfolding upon interaction with RESV was estimated using the algorithm for secondary structure determination implemented in the BeStSel webserver [55,56,57]. All fits had normalized root mean square deviations (NRMSD) < 0.012, confirming the reliability of the predicted secondary structures [58].

#### 4.2.2. Differential Scanning Microcalorimetry

The μDSC measurements were performed in aqueous alkaline solution at pH 11 and low ethanol concentration (1%, *v*/*v*). These conditions are necessary to ensure the solubility of zein while avoiding the negative effects produced by the high volatility of ethanol at elevated temperatures (bubble formation, cell instability, increased internal pressure in sealed systems, thermal signal distortion, baseline instability).

The thermal stability of zein solutions (6.3 × 10^−4^ M), in the absence and in the presence of RESV at RESV:zein molar ratios of 1:1 and 4:1, was investigated using a μDSC7 evo calorimeter (Setaram, Caluire, France). Solutions were heated from 298 K to 363 K with a scan rate of 1 K min^−1^, using an equilibration time of 30 min at the starting temperature. The data acquisition and processing were performed by Calisto v2.16 software using a linear baseline. The exothermic μDSC signals obtained correspond to zein aggregation, and the thermodynamic parameters (aggregation temperature, T_agg_, and enthalpy change, ΔH_agg_) were determined.

Although the solution studies, circular dichroism and μDSC, are performed under different experimental conditions, the literature data confirm that the native structure of the protein is not significantly altered [30], and the binding behavior of RESV and other lipophilic polyphenols remains consistent [30,43]. The main interactions between zein and RESV, hydrophobic and hydrogen bonding, do not change across these solvent conditions. Therefore, the behavior observed in solution can be reliably linked to the structure of the final gel.

#### 4.2.3. Molecular Docking

Zein Z3625 from Sigma-Aldrich is associated with the UniProt [59] accession number P04700 that corresponds to an α-zein structure with 22 kDa molecular weight and 263 amino acid residues, encoded by the z1C2 gene in *Zea mays*. As the producer specifies that this product is in fact a mixture of ~35% α-zein, β-zein (accession number P06673) and γ-zein (accession number Q548E9), the corresponding PDB structures of all three zein isoforms, representing single-chain models, were downloaded from the AlphaFold Protein Structure Database [60,61]. The structure of trans-RESV was optimized at the B3LYP/6-311G++ level of density functional theory, using the Gaussian03 software [62]. AutoDockTools 1.5.7. [63] was used to prepare the zein and RESV files for docking. The rotatable bonds of RESV were detected and assigned in order to obtain flexible conformers that interact with zein. To identify the amino acid residues of zein susceptible to interaction with RESV, molecular docking of RESV to the rigid protein was performed in AutoDock Vina 1.1.2 [64], using a population size of 150 and allowing up to 2,500,000 energy evaluations. The grid size was configured to cover the entire protein, with a grid spacing of 1 Å and dimensions of 78 Å × 70 Å × 70 Å, centered at coordinates −10, −10, −10 along the x, y, and z axes (in the coordinate system of the PDB file) for α-zein. The grid size is 58 Å × 58 Å × 60 Å, centered at coordinates 0, 0, 0 along the x, y, and z axes for β-zein, and 102 Å × 72 Å × 80 Å, centered at coordinates 10, 0, −5 along the x, y, and z axes for γ- zein. The zein–RESV pair with the lowest interaction energy was selected to evaluate the nature of the intermolecular forces, using BIOVIA Discovery Studio 2019 [65].

#### 4.2.4. Preparation of Zein and RESV:Zein Gels

**Figure 9:**
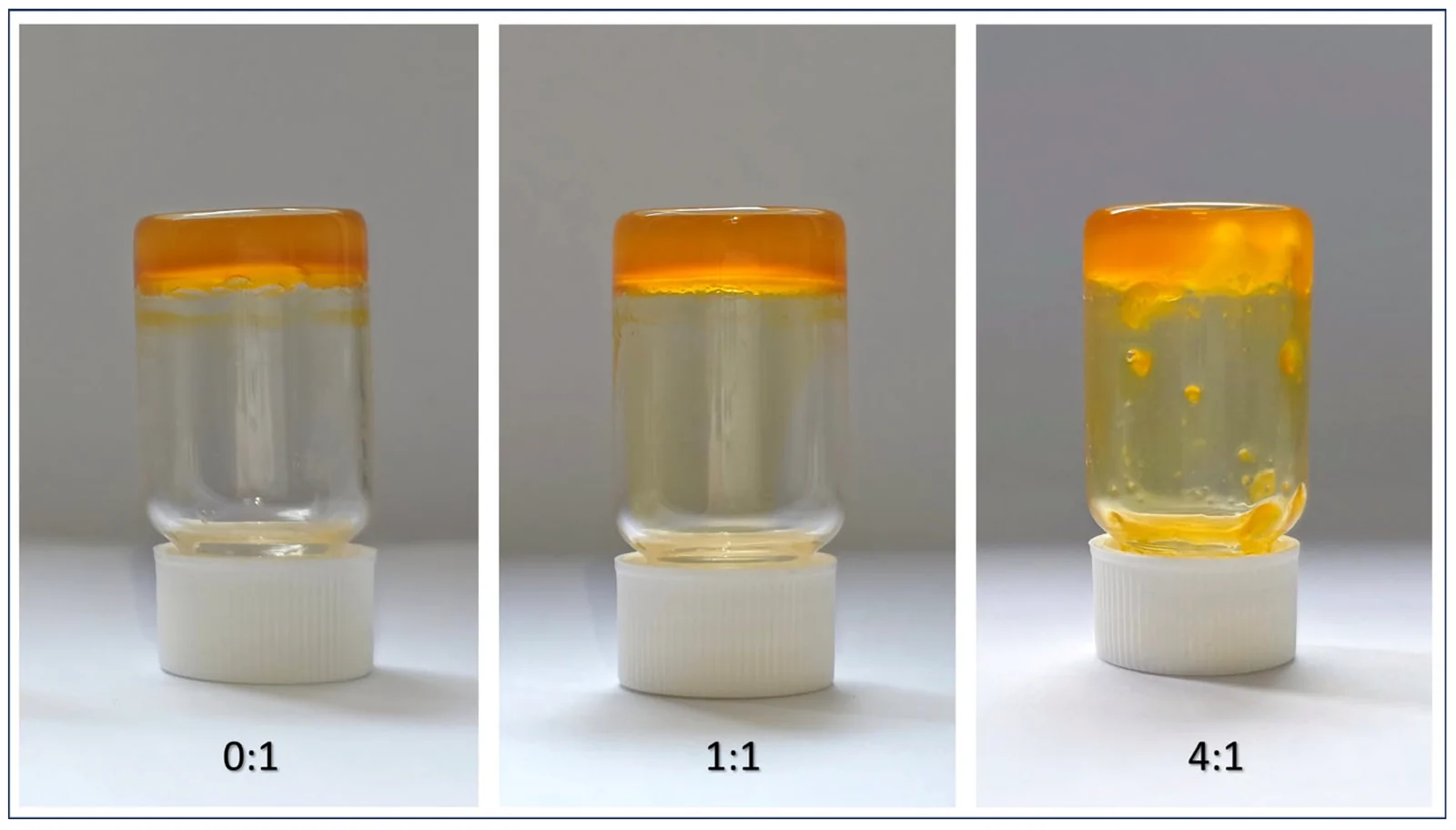
Samples prepared at RESV:zein molar ratios of 0:1, 1:1 and 4:1.

Zein gel was prepared by dissolving zein powder at a concentration of 1.5 × 10^−2^ M (33% *w*/*v*) in 80% (*v*/*v*) aqueous ethanol. The encapsulation study of RESV within a preformed zein gel (Figure S4 in the Supplementary Material and discussion therein) revealed a low encapsulation efficiency (16.50%) due to progressive solubilization of the zein matrix. Considering this, in order to obtain RESV:zein gels containing the active compound at concentrations corresponding to RESV:zein molar ratios of 1:1 and 4:1, RESV was dissolved in ethanol during the preparation of the zein gel. The solutions were continuously stirred at 1500 rpm and room temperature for 4 h until gelation occurred. Gel formation was preliminarily checked by the tube inversion method (Figure 9).

#### 4.2.5. Infrared Spectroscopy

The FTIR spectra of zein, RESV:zein 1:1 and RESV:zein 4:1 dried gel samples, and of zein and RESV raw commercial powders, were examined with a Nicolet i-S10 FT-IR spectrophotometer in ATR mode (Thermo Scientific, Waltham, MA, USA). The spectra were recorded over the 4000–525 cm^−1^ range using a spectral resolution of 4 cm^−1^, with 32 scans. To estimate the secondary structure of zein, the band in the amide I region (1700–1600 cm^−1^) was deconvoluted into the minimum number of Gaussian components. For this, five minima were selected from the second derivative of the FTIR spectra, as shown in Figure S5 for zein powder (1683, 1668, 1652, 1635 and 1621 cm^−1^), and used as input band positions [66,67]. Coefficients of determination and fit standard errors were >0.99 and <0.002, respectively.

#### 4.2.6. Fluorescence Spectroscopy

The steady-state fluorescence spectra of zein, RESV:zein 1:1 and RESV:zein 4:1 gel samples were recorded on a JASCO FP-8250 spectrofluorometer (JASCO Co., Ltd., Kyoto, Japan) equipped with an FPA-810 accessory for solid samples. Front-face fluorescence measurements (incident excitation angle of 30°) were performed at 298 K, using an excitation wavelength of 280 nm.

#### 4.2.7. Rheological Measurements

Rheological measurements were performed on a Kinexus PRO rheometer (Malvern Panalytical, Worcestershire, UK) coupled to a Julabo CF41 cryo-compact circulator (JULABO GmbH, Seelbach, Germany) to maintain the temperature at 298 K. The samples were placed between the two geometries with a gap of 0.6 mm, the upper geometry having a diameter of 20 mm and a cone angle of 4°. Amplitude sweep measurements were performed at 1 Hz, with the complex shear stress ranging from 1 to 1000 Pa for zein and RESV:zein 1:1, and from 0.2 to 20 Pa for RESV:zein 4:1. The viscoelastic characteristics of the samples were analyzed by a frequency sweep test ranging from 0.2 to 100 Hz at 10 Pa for zein and RESV:zein 1:1, while for RESV:zein 4:1, the applied shear stress was 1 Pa, recorded within the frequency range of 0.2 to 20 Hz.

#### 4.2.8. Resveratrol Release from Zein Gel

The release profiles of RESV from the zein gels prepared at RESV:zein 1:1 and 4:1 molar ratios were evaluated in 20 mM phosphate-buffered solution at pH 7.4 and normal physiological temperature (310 K), using a Spectra/Por dialysis cellulose membrane (Spectrum Laboratories Inc., Eindhoven, The Netherlands) with 6–8 kDa molecular weight cut-off. The release was monitored at 321 nm using a Carry Varian 100 Bio spectrophotometer (Agilent Technologies UK Ltd., Workingham, UK). No interference from the zein matrix was recorded.

To evaluate the mechanism of RESV release from the zein gel matrices (initial phase), the experimental data were fitted to the first-order kinetic model (Equation (1)) [68]:

(1) $$F_{t}=F_{\infty }(1-e^{-kt})$$

where *F_t_* is the cumulative percentage release up to time *t*, *k* is the first-order release rate constant, and *F_∞_* is the maximum percentage of RESV available for release. The release half-life, representing the time required to release 50% of the RESV available for release, was calculated as *t*_1/2_ = ln2/*k*.

The Weibull model [47], described by Equation (2), was used to fit the entire release process, indicating that the RESV release from zein gels is controlled by a combined effect of diffusion and matrix reorganization rather than by a single dominant transport mechanism.

(2) $$F_{t}=F_{\infty }(1-e^{-\frac{t^{\beta }}{\tau }})$$

In Equation (2), *F_t_* is the cumulative percentage release of RESV up to time *t*, *F_∞_* is the maximum cumulative percentage release of RESV, and τ and β are constants related to the specific release mechanism.

In Equations (1) and (2), as in the Higuchi and Korsmeyer–Peppas equations presented in the Supplementary Material, *F_∞_* is an adjustable parameter, determined by the best fit. The expression given in Equation (2) corresponds to the zero-lag time of the Weibull model.

## Data Availability

The original contributions presented in this study are included in the article. Further inquiries can be directed to the corresponding author.

## Supplementary Materials

The following supporting information can be downloaded at https://www.mdpi.com/article/10.3390/gels12080740/s1: Figure S1: The UV-Vis absorption spectra of zein (10^−5^ M) and RESV (10^−5^ M) in 80% (*v*/*v*) aqueous ethanol. In the zein spectrum, the most intense band (~280 nm) arises from the aromatic amino acid residues in the protein chain, specifically tyrosine. The band at 320 nm originates from dityrosine, which is naturally present within the protein and may increase in content during zein processing. The bands in the 400–500 nm spectral region (inset) arise from carotenoid pigments co-extracted with zein; Figure S2: The initial release phase (0–5 h) profile of the 1:1 RESV:zein gel, analyzed with (A) Korsmeyer–Peppas and (B) Higuchi models; Figure S3: The initial release phase (0–5 h) profile of the 4:1 RESV:zein gel, analyzed with (A) Korsmeyer–Peppas and (B) Higuchi models; Figure S4: Change in absorbance at 321 nm as a function of time, showing the encapsulation of RESV and the progressive dissolution of the zein gel matrix; Figure S5: Infrared spectrum of zein powder in the amide I region. The upper and lower figures represent the curve-fitted spectrum and the second derivative spectrum, respectively; Table S1: Infrared spectral deconvolution results, including peak positions, band areas, coefficients of determination and fit standard errors.
