# Supplementary material for: Understanding the Effect of Zein–Resveratrol Interactions on the Mechanical and Functional Properties of Zein Gels
Source: Gels. 2026 Aug 18;12(8):740. doi: 10.3390/gels12080740 (PMC13511865; doi:10.3390/gels12080740)
Supplement: Supplementary file 1 [file gels-12-00740-s001.zip › gels-4438886-supplementary.pdf]

Supplementary Materials

Understanding the Effect of Zein–Resveratrol Interactions on the Mechanical and Functional Properties of Zein Gels

Iulia Matei, Alexandra Busuioc, Ludmila Aricov, Anca Ruxandra Leonties, Vlad Tudor Popa and Aurica Precupas *

"Ilie Murgulescu" Institute of Physical Chemistry of the Romanian Academy, 202 Splaiul Independentei,
060021 Bucharest, Romania; iuliamatei@icf.ro (I.M.); alexandra.busuioc01@yahoo.com (A.B.); laricov@icf.ro (L.A.); aleonties@icf.ro (A.R.L.); vtpopa@icf.ro (V.T.P.)
* Correspondence: aprecupas@icf.ro

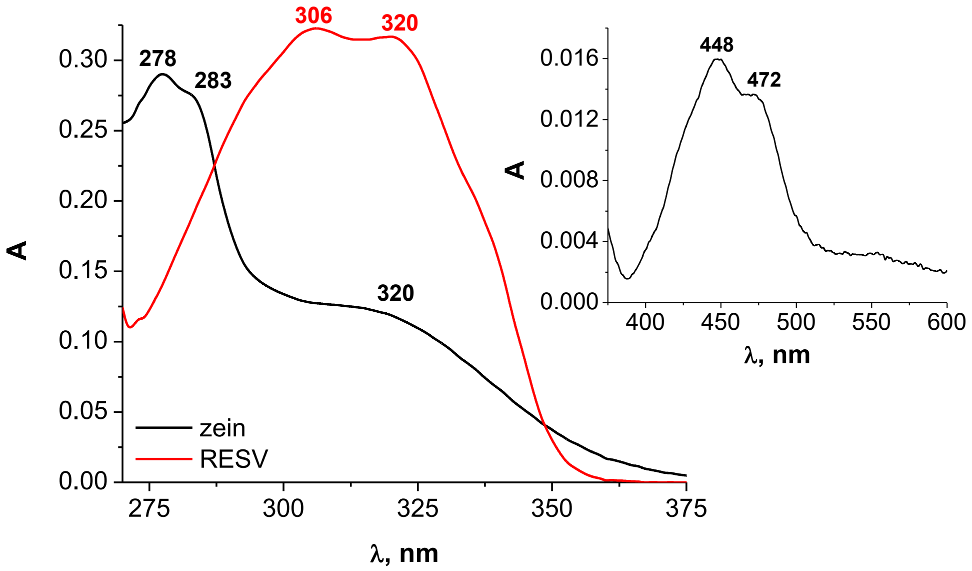

Figure S1. The UV-Vis absorption spectra of zein (10^{-5} M) and RESV (10^{-5} M) in 80% (v/v) aqueous ethanol. In the zein spectrum, the most intense band (~280 nm) arises from the aromatic amino acid residues in the protein chain, specifically tyrosine. The band at 320 nm originates from dityrosine, which is naturally present within the protein and may increase in content during zein processing. The bands in the 400–500 nm spectral region (inset) arise from carotenoid pigments co-extracted with zein.

Resveratrol release from zein gel

The Higuchi:

$$F_t = k_H t^{0.5} \text{ with } F_\infty \text{ embodied in the apparent release constant, } k_H,$$

and Korsmeyer–Peppas:

$$F_t = k_{KP} t^n \text{with } F_\infty \text{ embodied in the apparent release constant, } k_{KP},$$

models have been applied to early-stage diffusion processes, as presented in reference 47. Here, we use these models to analyze the release profiles of RESV:zein gels for the initial release phase (0–5 h, corresponding to $F_t/F_\infty \leq 0.6$ of the total accessible RESV). The resulting parameters are presented in Figures S2 and S3 for the 1:1 and 4:1 RESV:zein gels, respectively.

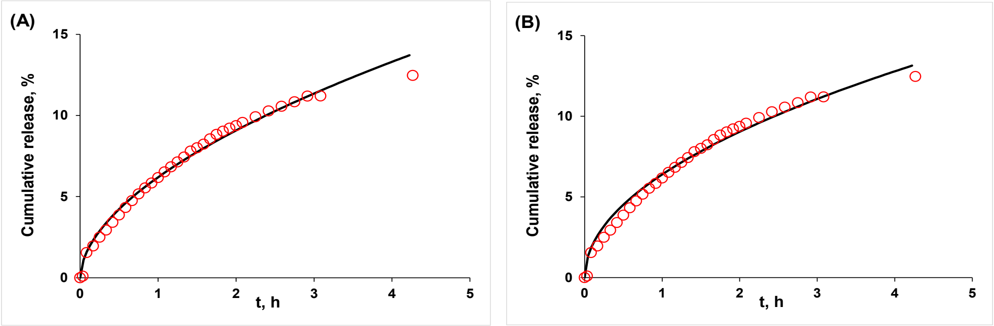

Figure S2. The initial release phase (0–5 h) profile of the 1:1 RESV:zein gel, analyzed with (A) Korsmeyer–Peppas and (B) Higuchi models.

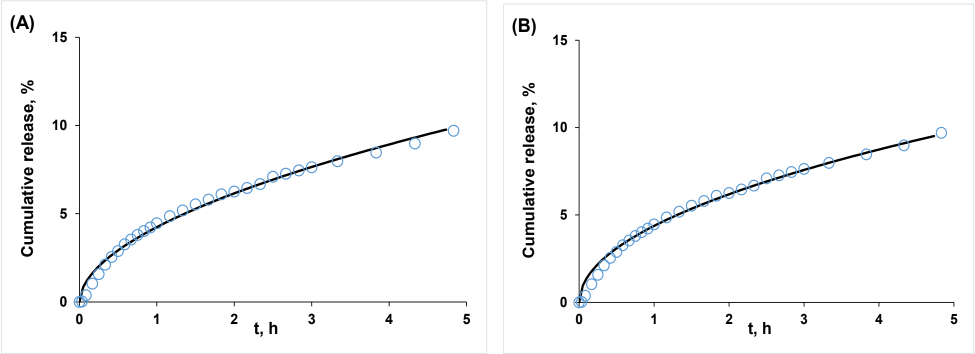

Figure S3. The initial release phase (0–5 h) profile of the 4:1 RESV:zein gel, analyzed with (A) Korsmeyer–Peppas and (B) Higuchi models.

For the 1:1 RESV:zein gel, the higher fit accuracy obtained with the Korsmeyer–Peppas model (R^2 = 0.987, k_{KP} = 6.19±0.08% h^{-n}, n = 0.55±0.01) confirms that the transport mechanism involves RESV diffusion and water-induced zein matrix compaction. The analysis using the Higuchi model yielded a dissolution constant k_H = 6.39±0.06% $h^{-0.5}$ and a high coefficient of determination (R^2 = 0.982). This result confirms that diffusion is the primary driving force during the initial hours of delivery, before the water-induced matrix compaction.

For the 4:1 RESV:zein gel, the Korsmeyer–Peppas model reveals a kinetic constant k_{KP} = 4.24±0.07% h^{-n} and a release exponent n = 0.53±0.01 (R^2=0.989). This indicates that RESV release is not restricted to simple concentration-driven diffusion, but rather involves RESV transport coupled with water-induced structural reorganization of the zein network. While the early-stage kinetics maintain a high linear correlation with the Higuchi model (k_H = 4.37±0.004% $h^{-0.5}$, R^2=0.986) due to dominant initial diffusion, the value of the Korsmeyer–Peppas exponent (n = 0.53) confirms that matrix compaction influences the RESV release.

Resveratrol encapsulation in zein gel

For the RESV encapsulation study, 0.123 g of zein gel was immersed in 7 mL 2×10^{-5} M RESV solution prepared in 80% (v/v) aqueous ethanol. The amount of encapsulated RESV was determined spectrophotometrically by measuring the absorbance of the solution at 321 nm over time. The absorbance at 321 nm from a blank sample prepared by immersing 0.123 g of zein gel in 7 mL of 80% aqueous ethanol was subtracted at each time interval to account for the progressive dissolution of zein from the gel matrix. The encapsulation efficiency (EE%), which quantifies the percentage of the initially added RESV that is entrapped within the gel network, and the loading capacity of RESV were calculated using Equations S1 and S2, respectively:

$$\mathrm{EE\%} = \frac{\mathrm{C_0 - Ce}}{\mathrm{C_0}} \times 100 \qquad \text{(S1)}$$

$$\text{loading capacity}\left(\frac{\mathrm{mg}}{\mathrm{g}}\right) = \frac{\mathrm{C_0\ (mg)} - C_e\ \mathrm{(mg)}}{\text{amount of zein gel (g)}} \qquad \text{(S2)}$$

where C_0 is the initial molar concentration of RESV at t = 0 min and C_e is the residual molar concentration of free RESV in solution at equilibrium.

Within the first minute, the RESV absorbance decreased sharply from an initial value of 0.651 (t = 0 min), as can be seen in Figure S4. Subsequently, a stable plateau was maintained up to 25 min, with a mean absorbance value of 0.544. This rapid absorbance decrease indicates an almost instantaneous entrapment of the polyphenol, yielding a calculated encapsulation efficiency of 16.50% and a loading capacity of 0.2074 mg/g.

It is important to note that the 80% ethanol solution acts as a solvent that progressively solubilizes the pre-formed zein networks. This matrix dissolution, combined with limited polyphenol diffusion, explains why adding RESV directly onto a pre-formed zein gel resulted in low encapsulation efficiency. To overcome this limitation, the 1:1 and 4:1 RESV:zein gels were prepared by dissolving the zein powder in an 80% ethanol solution containing RESV at the required concentration, followed by continuous stirring for 4 h at room temperature. This extended equilibration period ensures ample time for the complete dissolution of zein and interaction with RESV before gelation occurs.

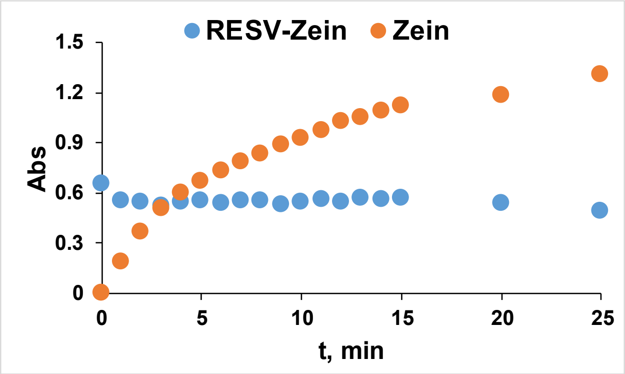

Figure S4. Change in absorbance at 321 nm as a function of time, showing the encapsulation of RESV and the progressive dissolution of the zein gel matrix.

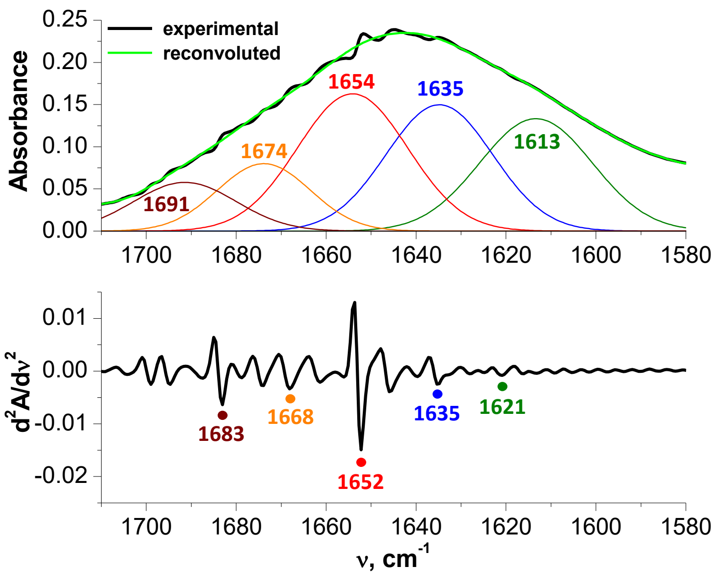

Figure S5. Infrared spectrum of zein powder in the amide I region. The upper and lower figures represent the curve-fitted spectrum and the second derivative spectrum, respectively.

Table S1. Infrared spectral deconvolution results, including peak positions, band areas, coefficients of determination and fit standard errors

Sample	ν, cm^{-1}	Area, %	R^2	Fit Std. Err.
zein powder	1613.3	24.1	0.998	0.0023
	1634.8	25.6		
	1654.1	28.8		
	1673.8	11.9		
	1691.4	9.5		
zein gel	1617.0	25.3	0.999	0.0019
	1634.4	32.6		
	1652.2	25.9		
	1673.4	8.8		
	1689.0	7.4		
RESV:zein 1:1 gel	1617.5	22.6	0.998	0.0014
	1632.4	31.6		
	1651.4	22.4		
	1672.5	15.2		
	1692.1	8.3		
RESV:zein 4:1 gel	1620.5	28.7	0.999	0.0025
	1634.6	29.7		
	1652.8	21.8		
	1671.1	10.3		
	1685.7	9.5		
